# Forgotten Contributors in the Brick Sector in Nepal

**DOI:** 10.3390/ijerph18126479

**Published:** 2021-06-16

**Authors:** Sugat B. Bajracharya, Kamala Gurung, Luja Mathema, Sanjay Sharma, Arabinda Mishra

**Affiliations:** International Centre for Integrated Mountain Development, Lalitpur, G.P.O. Box 3226, Kathmandu 44700, Nepal; Kamala.Gurung@icimod.org (K.G.); mathemadayal91@gmail.com (L.M.); adhikari.sd@gmail.com (S.S.); Arabinda.Mishra@icimod.org (A.M.)

**Keywords:** brick sector in Nepal, informal sector, decent working and living conditions, rapid needs assessment, informal workers

## Abstract

Brick making in Nepal is an informal sector that is still highly labor intensive. It employs transient workers who are extremely marginalized and exposed to poor working and living conditions. This study assesses the working and living conditions of male and female brick workers and their children and looks to address the main issues and challenges to promote decent living and working conditions in the brick factories through action research. A rapid needs assessment was conducted to take stock of the working and living conditions of male and female workers across five provinces in Nepal. Subsequently, selected pilot interventions through stakeholder consultations were initiated to address these issues and challenges. There are a host of challenges faced by these workers in the factories, with the main issues being occupational health and safety and childcare/education for workers’ children. The study suggests that the working and living conditions of the workers can be improved by incentivizing the brick entrepreneurs to invest in them, thus creating a more productive workforce. Moreover, the findings from the pilot interventions can help in the design of effective solutions.

## 1. Introduction

The brick sector is an integral part of urban development in South Asia, which employs millions of people [1]. The region is characterized by unprecedented urbanization coupled with economic growth [2]. Bangladesh, India, Nepal, and Pakistan account for 20% of global production [2]. Moreover, three countries (Bangladesh, India, and Nepal) are responsible for more than 90% of brick production in the South Asia region [2]. In the case of Nepal, the construction industry contributes 10–11% to the national GDP as per the reporting from Federation of Contractors’ Association of Nepal (FCAN). In relation to brick production, there were approximately 1600 brick factories with an annual production of 5 billion bricks as per estimates from Federation of Nepal Brick Industries (FNBI) in 2013 [2]. Nepal saw a meteoric rise in demand for bricks following the 2015 Earthquake, which required extensive post-earthquake reconstruction work. The demand for bricks was estimated to increase to 12 billion bricks annually owing to this [3]. This resulted in employment generation through the increase in brick factories to meet this demand.

Around 300,000 male and female workers were estimated to be engaged in brick production in Nepal in 2019 [4], with the majority of the workplace comprising migrants from marginalized and highly vulnerable social groups. These brick factories tend to fall under the umbrella of the informal sector due to the seasonality of the work and the use of informal low-skilled labor. The informality of the labor and the seasonality of the brick sector means that it attracts transient workers from different parts of the country as well as from India. The majority of male and female workers, both as families and individually, come from Province 5 (mainly from Dang, Banke, Bardiya, East Rukum districts), Province 6 (mainly from Salyan, Dailekh, Kalikot, West Rukum districts), and Province 7 (mainly from Kailali, Doti, Kanchanpur districts). Aside from these provinces, there are also workers who come from other parts of Nepal, such as Chitwan and other districts in the Terai region. In the case of India, the majority of male and female workers come from Uttar Pradesh, Bihar, and West Bengal on a seasonal basis. Be it workers from different parts of Nepal or India, the majority are affected by multidimensional poverty that is influenced by social, economic, political, and cultural drivers [5]. Their major livelihood options are nonfarm and farm work activities (such as wage labor) [6].

The brick sector is dominated by these seasonal migrant laborers who live at the factories during the brick season and move back to their homes during the off-season. Despite heavy investments in the sector, these workers do not have access to any form of social protection due to the informal nature of their work and, as a result, do not receive social welfare benefits or protection [2]. The lack of social protection leaves workers in an extremely vulnerable position as they are low skilled and are earning their living day by day. Workers in the sector are affected by a multitude of vulnerabilities when it comes to their working and living conditions. As is typical for many informal sector workers, they are also affected by ‘poor living conditions and indivisible social and economic rights’ [7].

Therefore, in order to address the situation of these marginalized workers, it is vital to understand the issues and take appropriate measures to incentivize the brick entrepreneurs in taking steps to improve the working and living conditions of all the workers involved in the production process. This paper presents cases of pilot interventions, as part of an action research approach, that seek to improve the working and living conditions of male and female workers in brick factories with the goal of achieving a decent working and living environment. The aim of the research was twofold: to understand the issues faced by male and female workers and their children in relation to the working and living conditions in brick factories through a rapid needs assessment; and addressing the main issues and challenges by carrying out action research for selected pilot interventions.

## 2. Materials and Methods

### 2.1. Conceptual Framework

In order to achieve and give cohesion to the research aims, a conceptual framework was developed to create guidelines for the rapid needs assessment as well as the subsequent process of stakeholder consultations to design action research pilot interventions, which will lead to improved working and living conditions for the male and female workers and their children in brick factories.

**Figure 1:**
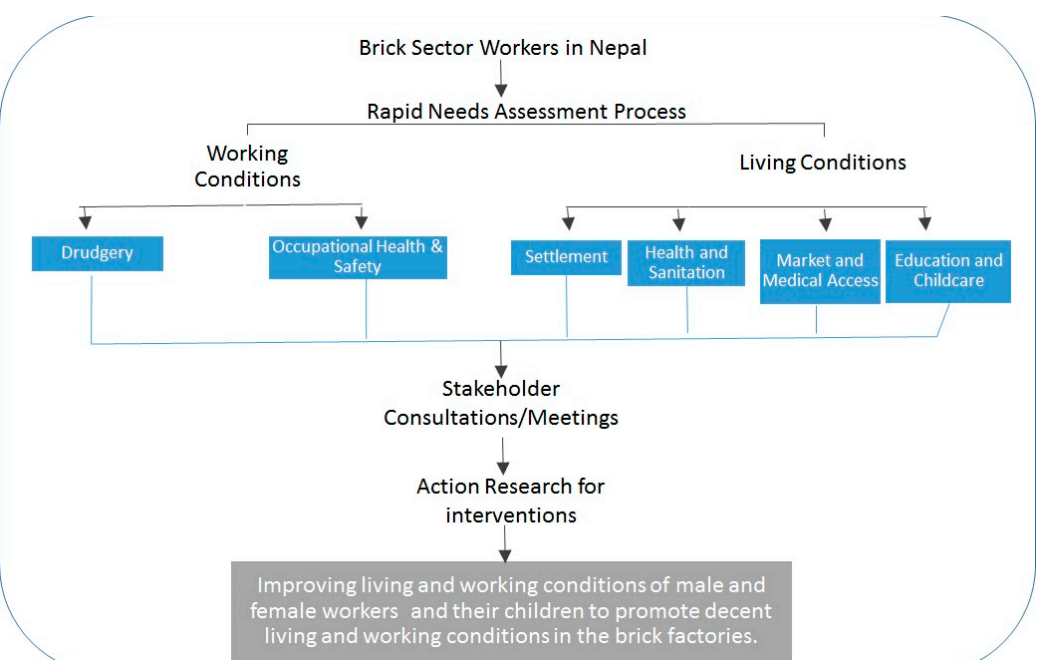
Conceptual framework.

Figure 1 illustrates and maps out the course of the research study.

Working and living conditions were looked into separately and considered as separate domains to capture their unique perspectives. Subsequently, we consider different factors under each domain to delve into the issues in more depth. Factors affecting the working conditions, such as drudgery and occupational health and safety were explored. Similarly, factors affecting the living conditions, such as the condition of the settlement areas, health and sanitation, accessibility to market and medical services, and the provision of education/childcare centers, were looked into. Eventually, we hope that by addressing the main issues and challenges faced by the workers relating to their working and living conditions, we can strive to achieve better productivity from the workers as a result of improved working and living conditions through selected pilot interventions.

### 2.2. Sampling and Site Selection

A purposive sample of brick factories was recruited for the purpose of this assessment. The provinces and factories were chosen in collaboration with the Federation of Nepal Brick Industries (FNBI). They were Province 1 (Morang district), Province 2 (Rautahat district), Province 3 (Dhading district), Province 5 (Rupandehi district), and Province 7 (Kanchanpur district). The provinces are mostly located in the southern Terai belt of Nepal with the exception of Dhading in Province 3. In each district, as per the recommendations of the district associations, three to four factories were visited for the assessment. The brick factories were chosen with the following criteria in mind: (1) location—by province as recommended by the FNBI team so as to capture provincial variations; (2) the scale and size of the brick factories (small, medium, large); (3) and technology applications (mechanization). The criteria were established in order to gain an insight into the working and living conditions in brick factories of various types across different locations. It also encompasses a range of brick factories, as different types of factories face different issues.

### 2.3. Methodological Framework

A rapid needs assessment process (RNAP) was selected as the preferred methodology due to its effectiveness in studying naturalistic settings and processes such as organizational practices and implementation in a time-efficient manner [8,9]. A particular methodological emphasis was placed on a time-sensitive team-based qualitative analysis process [10]. As our major focus was on designing and testing action-based pilot interventions, a process of information gathering that was thorough and time efficient was needed to ensure there was adequate time for discussions and the design and implementation of appropriate pilot interventions. The following steps as given in Table 1 were followed for the RNAP:

### 2.4. Data/Information Collection

The RNAPs, which was composed of a qualitative approach in the form of focused group discussions (FGDs), were conducted with each occupation group in the brick processing chain with the use of open- and closed-ended questions. The questions largely dealt with the working and living conditions in the factories from the workers’ perspectives. The workers were asked about the availability of basic services in the factories, such as drinking water, sanitation, health access, childcare centers, conditions of the settlement area, etc. The team decided to divide tasks and split up during the discussions in order to efficiently collect the data/information and understand the different perspectives and issues faced by men and women and to ensure their comfort. Tasks were split within the group according to time constraints and to ensure privacy when speaking to women in the groups about their health and other personal issues. Groups varied in size from 5–15 workers due to the working hours and work load. The groups of workers comprised workers from each component in the brick factory, from brick molders to transporters to the firepersons. At least one or two groups of women workers were sought from each factory to obtain their perspectives. The majority were married women who had come with their families. They primarily worked as transporters and molders.

The data/information collection schedule is illustrated in Table 2.

## 3. Results and Discussion

### 3.1. Rapid Needs Assessment Findings

The first part of the assessment focused on learning about the nature of the work and the working and living conditions in the brick factories. In order to understand the daily working situation of the workers, we followed up with them on their working hours to obtain a profile of their typical working day and an insight into the drudgery involved. Our observations and interactions show that the working hours during a typical day vary according to the nature of the tasks that the male and female workers are involved in. For instance, the payment for molders and transporters is largely dependent on the number of bricks they are able to mold or transport. Hence, the number of hours they put in is dictated by this. As a result of the low per piece rate, molders and stackers are required to put in more hours to earn enough. On the contrary, stackers and firepersons tend to have a set schedule dictated by the needs and operation of the factory. A detailed breakdown of their work hours is given in Table 3.

Similarly, there are a host of issues relating to the working and living conditions of the workers, from inadequate adherence to occupational health and safety protocols to a lack of basic services and sanitation in the workplace and settlement areas. Overall, the assessment findings illustrated two major issues. First, there were prevalent health and safety issues in the factories. There was a need for awareness among the male and female workers regarding good occupational safety practices. In addition to this, accidental and health insurance services were direly needed. Secondly, there was a need to address childcare and education issues surrounding the workers’ children in the factories. Details of the findings are as follows.

#### 3.1.1. Occupational Health and Safety 

During the assessment, we observed that the workers from all stages of the brick production chain tend to be exposed to at least some form of occupational risk in their daily schedules. Be it in the form of physically demanding work that is straining or working in dangerous locations with massively unhealthy exposure to dust and heat, male and female workers face tough occupational health and safety challenges in their workplace. The firepersons, stackers, and transporters tend to bear a higher risk during their work. The firepersons have to work on a daily basis surrounded by high temperatures that tend to be physically straining. The stackers face a risk of injury as they climb the brick piles while stacking unbaked bricks and dismantling the baked bricks afterwards. Moreover, they are constantly exposed to dust during the stacking/dismantling process, which is detrimental to respiratory health. Similarly, the transporters face an increased risk of back and neck injuries and respiratory illnesses from exposure to dust when carrying/transporting heavy loads of bricks to and fro. Molders, on the other hand, faced risks pertaining to continuous work in a set position for long hours leading to back and leg pain.

Our observation shows that compliance to occupational health and safety precautions and measures was very low among most male and female workers in the factories. We found that many workers did not wear masks, gloves, or closed shoes despite being provided with these items by the owners. The masks were seen as uncomfortable while working, the gloves were torn easily and hindered efficient work, and the shoes were not considered to be comfortable for wearing during hot weather. Many women workers tended to use their own shawls to cover their heads and faces to protect themselves from dust exposure. There were instances in some factories where the occupational safety materials were used diligently throughout the brick-making process. However, these were limited to a few places.

The workers were generally provided with basic medical or health services, such as first aid kits, in the factories, and visits to a local health post or a nearby hospital were required for other emergency medical issues. However, there were very few or no provisions of insurance relating to accidental injuries or health-related issues.

#### 3.1.2. Childcare and Education for Workers’ Children

Young children are often part of the family of the workers who come to the factories to work. To cater for this, there were centers providing education and childcare for children in the brick factories in some of the provinces, with a variation in the services provided. For example, a few factories in Dhading had childcare centers for young children, with support from Better Brick Nepal (BBN). While some young children went to the childcare centers, in other cases, the children in the factories were sent to local schools.

Moreover, a factory in Biratnagar employed a female Bengali teacher to cater to non-Nepali-speaking young children in their care center. There were no day-care centers in Rautahat or Kanchanpur districts. While some factories have day-care centers and others associate with schools, the underlying problem is that there is a lack of quality and continuity of education for children, who have to spend 6 months in their homes and another 6 months in locations where the factories are located. As a result, local government schools often refuse to take in children from the factories due to the difficulty in rehabilitating children who have missed part of the curriculum.

### 3.2. Stakeholder Consultations to Decide on Pilot Interventions

The consultations with various stakeholders were organized to share the findings from the assessment and to discuss possible pilot interventions that address the main issues. The stakeholders were selected based on their areas of expertise and experience of piloting and implementing viable solutions on the ground. They included brick entrepreneurs, members of the FNBI, and private sector companies, such as Shikhar Insurance (for the possibility of insurance for workers in cases of accidents), Ecoprise (for solar lighting in the factories), and OLE Nepal (children’s education/childcare).

There were discussions relating to the feasibility of the various pilot solutions that were suggested, of which, education for workers’ children, health/accidental insurance, workplace safety with adequate lighting, and occupational health and safety awareness were the main subjects discussed. Solutions relating to solar powered lighting to light the working spaces of molders, who work during early morning hours in the factories, were deemed impractical due to the investment required, the temporary nature of the factory establishment, and the brick-making season coinciding with the months that receive insufficient sunlight to properly power the solar lights.

The discussions during the stakeholder consultations led to the design of three viable pilot interventions that were feasible for testing/piloting, namely:

1. An ICT education pilot for children in a school in Dhading district; the distribution of early child development (ECD) materials in factories with childcare/day-care centers;
2. Accidental/medical insurance for brick workers;
3. Awareness posters in the factories relating to occupational health and safety (OHS) and Social code of conduct (COC).

### 3.3. Pilot Interventions for Action Research

The pilot interventions were carried out through action research, which allowed us to simultaneously take action, perform research, and reflect on the effects of these interventions. It is important to note that the pilot interventions are on-going and their progress so far is detailed below: 

#### 3.3.1. ICT-Based Education Pilot Intervention in Shree Kalika Basic School in Dhading District

OLE Nepal in partnership with ICIMOD launched an e-learning program at Shree Kalika Basic School in Ward No. 4 of Dhunibesi Municipality in Dhading in 2019. This school runs classes from the ECD class to Class 4, where 60% of students are brick workers’ children. The program was started to achieve the dual objective of improving attendance among brick workers’ children and promoting interactive self-learning through ICT-based learning programs. The idea behind the program was to accommodate the children of brick workers and provide them with a self-learning platform that facilitates subject-specific, grade-specific national curriculum-based digital learning. This was set up in an attempt to bridge the gaps in the learning record of children of brick workers, who may have discontinuity in schooling due to the seasonal nature of their parents’ jobs. Moreover, the program also aimed to decrease the dropout rate of the children of brick workers, as they were more prone to do so.

The ICIMOD, in conjunction with the Brick Factory Association of Dhading, OLE Nepal, and the local government, was able to interact with the brick workers and the larger community to encourage participation and garner support to ensure sustainability of the program. OLE Nepal procured 25 laptops with interactive digital content called E-Paath, which contained interactive digital materials based on the national curriculum. In addition, ECD materials were also provided to the school. Teachers in the school were trained on the digital materials and ECD.

The record shows that there was a 10% increase in the overall enrolment of children in the school, from 78 students in 2019 to 86 students in 2020. Similarly, the enrolment of children of brick workers looked encouraging as it increased by 3% from 2019 to 2020. The feedback from the teachers suggest an improvement in attendance and increased interest from students as a result of the ICT-based learning tools. One of the positive influences of the program came in the form of interest and funds from the local municipality, which provided the school with necessary support in the form of furniture for the classrooms and midday meals. This liaison with the local municipality will provide an impetus to ensure the sustainability of the program beyond the project duration.

#### 3.3.2. Accidental/Medical Insurance for Brick Workers

Shikhar Insurance in collaboration with ICIMOD looked into the possibility of the provision of insurance for the brick workers to insure them against accidental injuries and medical issues during work. The rationale behind facilitating this initiative was to safeguard the workers as well as the owners from the risk of accidental injuries/health-related issues during work. At first, the insurance providers were reluctant to venture into this due to the informal and seasonal nature of the brick sector, which made the insurance provision slightly problematic.

Multiple rounds of discussions (meetings in Bhairahwa and Biratnagar) took place between the brick factory owners, the FNBI, and Shikhar Insurance to facilitate a provision of insurance for workers against accidental injuries and medical issues. The discussions resulted in the building of insurance packages for accidental insurance, medical expenses insurance, and brick factory insurance (insurance for the bricks and other assets of the factory).

The specifics and package details will be finalized in future consultations. The insurance board approved the drawing up of the policies. When the policies are operational, it will safeguard the brick workers from accidental injuries and medical costs incurred as a result of work.

#### 3.3.3. Occupational Health and Safety Awareness Posters and Social Code of Conduct (COC)

Raising awareness was considered to be of paramount importance as the workers’ awareness levels in relation to work health and safety were found to be very low as per the assessment findings. Compliance with occupational health and safety measures was seen to be lacking, with little care being given to the brick directives issued by the Department of Labor.

There is a need for a multipronged approach to deal with this issue. This must involve improving the awareness level of workers and factory owners, and convincing the factory owners to self-regulate the occupational health and safety measures. In order to increase the awareness levels of both factory workers and owners, awareness posters relating to occupational health and safety measures were distributed to the FNBI member factories with the direct participation of the factory owners themselves. The brick directives issued by the Department of Labor were deemed to be impractical and too broad (especially in relation to child labor and social security laws) for many brick factories, as the directives did not take stock of the realities on the ground. With this in mind, a social code of conduct document was drafted and agreed upon by the FNBI to regulate their member factories, with achievable and practical regulations that were customized for the brick sector. This was a culmination of a process involving meticulous discussions and a series of meetings on the provincial and general level.

In order to take this forward, an operational procedure document, i.e., a standard operational procedure (SOP), was prepared to initiate the implementation in the FNBI member factories. Initial efforts focused on implementing ‘model factories’ in different provinces in order to provide an example for other factories in the area to observe and replicate best practices.

## 4. Limitations of the Study

The study uses a qualitative methodology with focus group discussions as the primary data collection tool. Hence, detailed disaggregated quantitative data were not collected, as the main purpose of the assessment was to assess the situation of the working and living conditions of different groups of workers in the brick factories through discussions. Additionally, some of the interventions are on-going and this article presents the findings from them so far.

## 5. Conclusions and Way Forward

This action research study demonstrates that there is a need for a multipronged approach to tackle the issues in brick factories in terms of the provision of decent working and living conditions. On one hand, there is a need for sensitivity, awareness, and willingness from the factory owners to make amends, in order to improve the working and living conditions in their factories. On the other hand, there is a need for awareness among the male and female workers in relation to occupational health and safety measures that can contribute to creating and maintaining a decent and safe working/living environment. In addition to this, the successful initiation of pilot interventions indicate that the factory owners are willing to make improvements provided that there is a favorable and safe environment in which issues can be brought to the forefront and discussed to seek amicable solutions.

The study suggests that the working and living conditions of the male and female workers can be improved, which could have implications for brick production. On the other hand, factory owners are reluctant to invest in provisions for the working and living conditions of the workers due to the costs and effort involved. While many brick entrepreneurs consider investment in mechanization as a positive step to increase productivity and profits, they tend to forget the main contributors in this production process: the workers. As the brick sector is predominantly a labor-intensive sector, improving the working and living conditions in the factories not only benefits the workers, but it also benefits the owners through the retention of a productive and healthy workforce. This can incentivize the factory owners in investing in their workforce for a better return on their investment, while keeping the turnover to a minimum.

Moving forward, the reflections from the pilot interventions can help assess the effectiveness and scope of any improvements or upscaling. As these are on-going research interventions, the lessons learned from the pilot experiences are valuable for the proposal of effective interventions. The provision of insurance to workers and the use of a social code of conduct in the FNBI member factories are sure to positively impact the occupational health and safety of the workers. Similarly, the ICT-based program in the school of Dhading district provides an innovative way to increase enrolment, attendance, and bridge the gap in learning of brick workers’ children. However, the feasibility of upscaling is dependent on the capacity of schools to procure ICT laptops, arrange the necessary infrastructure, and manage the required funding. Therefore, to make it a sustainable venture, the involvement of the private and public sector is necessary. It is clear that any educational intervention to encourage increased enrolment and attendance of brick workers’ children requires the active participation of the local government, factory owners, and the school(s) in the region.

## Figures and Tables

**Figure 2:**
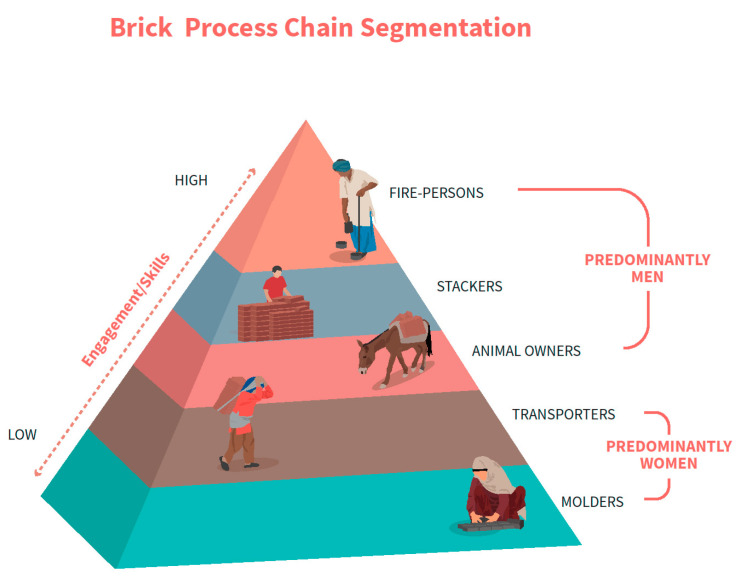
Brick process chain segmentation.

**Table 1:** Steps in the rapid needs assessment process.

Stage	Step
Design	Iteratively work with the internal team in establishing focus questions consisting of open-ended questions to enable exhaustive answers. Incorporate a conceptual framework to guide the field research and maintain the direction.
Data/information collection and analysis	3. Establish rapport with district/provincial liaisons via preparatory meetings by briefing them on the assessment. 4. Visit brick factories for qualitative data/information collection through focus group discussions with workers, observations, and interactions with brick entrepreneurs. 5. Through team discussions, compile the qualitative data into a chart developed to triangulate, compile, and develop emerging themes from the findings.
Validation and reporting	6. Write up the findings from each factory to reflect interactions with each group of workers (molders, transporters, stackers, firemen).

**Table 2:** Brick factories visited for the study.

Province	District	Brick Factories Visited	Estimated No. of Workers Surveyed	Dates Visited
1	Biratnagar	Anand ACC	20 15	18–21 April 2018
2	Rautahat	Ram–Janaki Star Special B1	20 15–20 15 22	15–18 April 2018
3	Dhading	Raktakali Kalika Everest Shivam	20 15 15 15	26–28 March 2018
5	Rupandehi	Ganesh AK Siddhartha	15 15–20 15–20	20–23 May 2018
7	Kanchanpur	Bajrangi Tirtha-sundari Shikshankhar Traditional Bengali Brick Factory	20 15 15 15–20	23–27 May 2018

Subsequently, multiple stakeholder meetings were conducted with various actors: ECOPRISE Pvt. Ltd., Open Learning Exchange (OLE) Nepal and Shikhar Insurance for Life and Health Insurance, including FNBI and entrepreneurs from the district level. The aim of these meetings was to gain insight into the issues and challenges of the male and female workers and to discuss ways to address them.

**Table 3:** Working hours and earnings of different categories of workers in the brick factories.

Brick Workers	Time	Description
Molders	(1) 2AM–6AM *Break from molding to cook food/look after children* (2) 8AM–11AM *Break from molding (in cases of no mechanization, clay is prepared by the molders)* (3) 5PM–7PM approximately	They mostly worked during the early morning hours. They took a break for their daily tasks in the afternoon. In most instances, they stopped molding during the day. Molding bricks in the midday heat has a negative effect on the quality of the brick as the heat causes the freshly molded bricks to crack (some molded during the day to earn more money). They earn USD 0.009–0.01 per brick molded. A family can make USD 6–21 a day by molding depending on the time taken for clay preparation.
Transporters	9AM–5PM with lunch break in between (time depends on individual due to rates). It was also found that transporting occurred when it was necessary to move the bricks.	The transporters worked throughout the day. In Rupandehi, Ganesh Brick Factory transporters had mini-trucks to transport green bricks, which helped decrease drudgery. In Biratnagar, the association implemented a rule saying transporters were not allowed to transport bricks on their head. They earn in the range of USD 2.14–2.8 per 100 bricks that they transport.
Stackers	9AM–5PM with lunch break in between (dependent on the schedule set in the brick factory)	Stackers work during the day whenever they are needed. This was considered ‘skilled labor’ as there was a certain level of skill needed to stack, especially in zig-zag factories, and climb the stacked bricks. Their earnings range from USD 164–191.3 per month.
Firepersons	Throughout the day and night	Fireworkers take shifts during the day in order to have a break from the heat of the fire. They earn a salary in the range of USD 150.3–205 per month.

In addition to this, our observations also show that there is a hierarchy among the workers according to their skill level and gender. Higher skilled workers tend to earn more and are involved in jobs like firing and stacking, while lower-skilled workers tend to be involved in jobs like transporting and molding. Women tend to be involved in jobs towards the lower end of the skills and income spectrum, working predominantly as molders and transporters. This is demonstrated in the brick process chain segmentation presented in Figure 2 below.

## Data Availability

Not applicable.
